# Implication of DNA methylation during lifestyle mediated weight loss

**DOI:** 10.3389/fendo.2023.1181002

**Published:** 2023-08-08

**Authors:** Samantha Aurich, Luise Müller, Peter Kovacs, Maria Keller

**Affiliations:** ^1^ Medical Department III - Endocrinology, Nephrology, Rheumatology, University of Leipzig Medical Center, Leipzig, Germany; ^2^ Helmholtz Institute for Metabolic, Obesity and Vascular Research (HI-MAG) of the Helmholtz Center Munich at the University of Leipzig and University Hospital Leipzig, Leipzig, Germany; ^3^ Deutsches Zentrum für Diabetesforschung e.V., Neuherberg, Germany

**Keywords:** overweight, obesity, weight loss, lifestyle, intervention, DNA-methylation, epigenetics

## Abstract

Over the past 50 years, the number of overweight/obese people increased significantly, making obesity a global public health challenge. Apart from rare monogenic forms, obesity is a multifactorial disease, most likely resulting from a concerted interaction of genetic, epigenetic and environmental factors. Although recent studies opened new avenues in elucidating the complex genetics behind obesity, the biological mechanisms contributing to individual’s risk to become obese are not yet fully understood. Non-genetic factors such as eating behaviour or physical activity are strong contributing factors for the onset of obesity. These factors may interact with genetic predispositions most likely *via* epigenetic mechanisms. Epigenome-wide association studies or methylome-wide association studies are measuring DNA methylation at single CpGs across thousands of genes and capture associations to obesity phenotypes such as BMI. However, they only represent a snapshot in the complex biological network and cannot distinguish between causes and consequences. Intervention studies are therefore a suitable method to control for confounding factors and to avoid possible sources of bias. In particular, intervention studies documenting changes in obesity-associated epigenetic markers during lifestyle driven weight loss, make an important contribution to a better understanding of epigenetic reprogramming in obesity. To investigate the impact of lifestyle in obesity state specific DNA methylation, especially concerning the development of new strategies for prevention and individual therapy, we reviewed 19 most recent human intervention studies. In summary, this review highlights the huge potential of targeted interventions to alter disease-associated epigenetic patterns. However, there is an urgent need for further robust and larger studies to identify the specific DNA methylation biomarkers which influence obesity.

## Introduction

Obesity has reached pandemic proportions in the last 50 years and represents a worldwide health challenge (1). People who are overweight or obese have a higher risk for numerous cardiometabolic diseases such as type 2 diabetes (T2D), dyslipidemia, stroke and hypertension (1). Beside some rare monogenic forms (2), obesity is a multifactorial disease driven by an interaction of polygenic predispositions and the exposure to obesogenic environmental factors. Various diets with different levels of macronutrients have been tested and compared to define successful weight loss strategies (3), however, responses to dietary changes are extremely individual. That is why personalized nutritional recommendations are becoming more and more important to meet individual needs. Lifestyle interventions such as increasing energy expenditure through intensive physical activity and lowering energy intake through various diets are important set points to control the disease. However, long-term success is often very individual and weight regain quite common (4). In order, to better understand this highly complex interplay, research into genetic and lifestyle-related factors that influence human metabolism, most likely mediated by epigenetic regulations, is inevitable. Future findings might contribute to better understanding of the high variability in individual's response to specific nutritional and physical activity interventions and may improve long-term therapy by personalisation (5).

Although epigenetics and its involvement in metabolic diseases still represents a young research field, it has significantly advanced during the last two decades. Epigenetic modifications include DNA methylation, histone modifications, and non-coding RNAs which are regulating cell-specific gene expression, cell differentiation, parental imprinting, X chromosome inactivation, and genomic stability and structure (6). DNA methylation is the most commonly studied modification and describes the methylation of the carbon 5 position within cytosine bases, resulting in 5-methylcytosine. This occurs predominantly at cytosines within CG dinucleotides ("CpG" sites) in mammalian genomes and is associated with gene silencing when occurring at gene promoters and enhancers (7). Epigenetic modifications are mediating between environmental and genetic factors - environmental changes can lead to epigenetic regulations, which in turn can affect gene activity (8). There are two different starting points to analyse epigenetic modifications in regard to obesity. On the one hand, epigenetic alterations might be causal for the development of obesity by inducing inappropriate expression or silencing of obesity-associated genes and regulatory sequences, leading to metabolic imbalances (9). On the other hand, epigenetic changes can also arise as a consequence of obesity and predispose obesity-associated co-morbidities such as T2D (10) or cancer (11, 12).

Epigenome-wide association studies (EWAS) represent a powerful tool for investigating associations between epigenetic markers and the obesity state defined by BMI or parameters of fat distribution, aiming to understand the molecular mechanisms underlying the disease risk (13). EWAS or methylome-wide association studies (MWAS) measure DNA methylations at single CpGs or regions containing CpGs across thousands of genes using genome-wide approaches based on techniques such as Illumina arrays or whole genome bisulfite sequencing and analyse potential associations with disease phenotypes. However, they only represent a snapshot in complex biological networks and cannot distinguish between causes and consequences. Intervention studies are therefore a suitable method to control confounding factors and avoid possible sources of bias. In particular, intervention studies which document changes in obesity-associated epigenetic markers during weight loss can make an important contribution to this research field. Peripheral blood is the most commonly used biospecimen for these intervention studies (14). This is due to its accessibility through a minimally invasive procedure and due to the fact that blood is often the only available source for a biomaterial. Additionally, some studies suggest that specific DNA methylation changes in blood may reflect pathological conditions in target organs that are not accessible by biopsy (15). However, while DNA methylation profiles in blood can summarize information about systemic exposures or diseases, they cannot specifically assess cells from a single organ or tissue (16). Studies use different techniques to control for the effect of cell heterogeneity and thus possible confusion (17, 18). These include various deconvolution techniques (19), which provide a framework for estimating the relative proportions of blood cell types. Of note, there are studies, which identified an epigenetic signature potentially mirroring the epigenetic regulation of obesity-related adipose tissue dysfunction. These studies provide a DNA methylation map in circulating leukocytes reflecting subcutaneous adipose tissue methylation pattern by comparing both tissues in patients with obesity vs normal-weight individuals (20–22).

Although corresponding research is advancing very fast, aiming to identify new markers allowing the development of new therapy options in obesity, the last systematic review was performed by Aronica et al. in 2017. The authors summarised candidate-based and genome-wide approaches analysing DNA methylation at baseline and at the endpoint of a weight loss intervention in overweight/obese subjects who were free of comorbidities such as hereditary diseases or cancer (8). They identified 25 longitudinal intervention studies over eight years (from 2008 to 2016), which examined either pre- vs. post-interventional (diet and/or exercise/ metabolic surgery interventions) DNA methylation changes or DNA methylation differences between patients with high and low response to the intervention. Back in 2017 the majority of studies were candidate gene approaches (*N*=16), while only nine used genome-wide data sets analysing either differentially methylated single CpG positions (DMPs) or differentially methylated regions (DMRs).

However, since 2017 numerous new studies in this field came up with improved interventional and analytical approaches, further highlighting lifestyle habits as therapeutic options and strongly contributing to our understanding of the mechanistic role of DNA methylation in obesity. In the present review, we focus on human interventional studies, which aim to reduce body weight in individuals with overweight or obesity and which are based on the main lifestyle parameters: diet and physical activity.

This review discusses the contribution of candidate-based and genome-wide DNA methylation studies in regard to lifestyle treatments, including a special focus on methylation age and *in-utero* studies. We discuss recent achievements in this young field of research and point out strengths and possible weaknesses. We finally wish to draw attention to the potential of this field for the development of new strategies for prevention and individualised therapies.

## Methods

### Screening and inclusion/exclusion criteria

**Figure 1:**
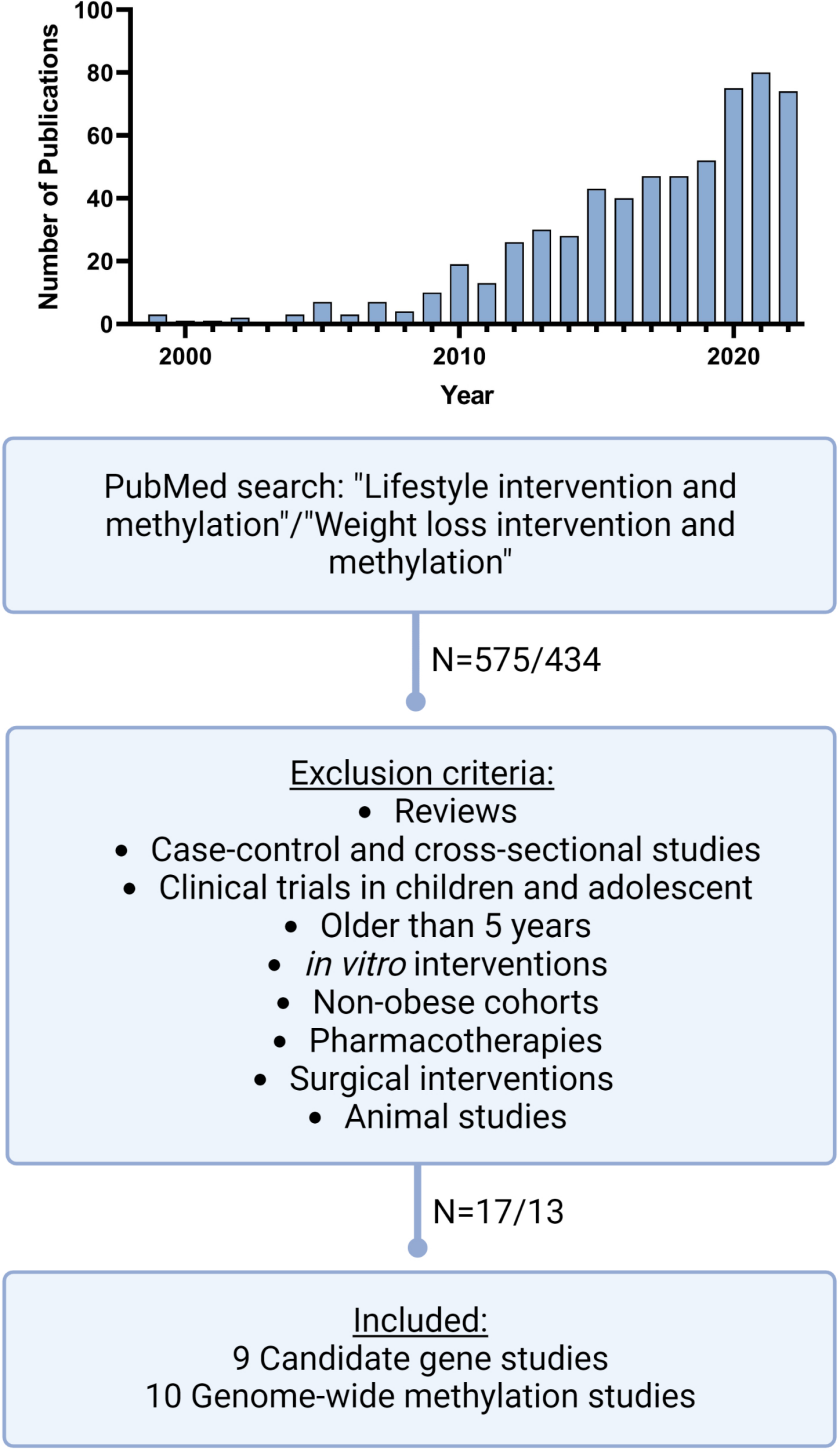
Literature search workflow. Schematic presentation of our literature search on PubMed including mash terms, exclusion criteria and publication numbers. In addition, the top figure shows the distribution of the numbers of publications during the recent years, with a sharp increase of publications matching our mash terms since 2010 but also the most significant increase in the last 3 years. (Created with Biorender.com).

We performed a PubMed search (dated 05.07.2023) as described in **Figure 1**, for studies published during the last five years (2018-2023) using the following mash terms: “lifestyle intervention and methylation” and “weight loss intervention and methylation”. We focused on longitudinal studies measuring either specific candidate loci, global or genome-wide DNA methylation at baseline and after finishing an individual lifestyle intervention designed to lose weight and/or improve general health conditions in subjects with overweight or obesity. Cross-sectional studies, case-control studies, longitudinal studies without intervention, interventional studies conducted *in vitro* only and in animals or subjects without overweight or obesity were excluded. We further excluded subjects undergoing pharmacotherapies, weight loss surgeries and those suffering from severe disease stages such as cancer. On the other hand, we included studies comparing responder vs. non- responder to respective lifestyle interventions. Furthermore, we focused exclusively on DNA methylation, excluding other epigenetic mechanisms such as histone modifications and noncoding RNAs.

## Results

### Current state of research

Our structured PubMed search revealed an eminent increase of studies during the last 5 years, dealing with DNA methylation under weight loss conditions. In the years before 2009, the number of publications was still in the single digits and rose sharply to 80 publications in 2021 (**Figure 1**). We identified nine new candidate gene and ten new genome-wide studies, which were performed between 2018 and 2023. All candidate gene approaches analysed changes in DNA methylation before and after an individual lifestyle intervention, while the identified ten genome-wide studies (**Table 1**) assessed either DNA methylation before and after weight loss (N=7) or compared responder vs. non-responder (N=3) after different lifestyle interventions. The majority of them used array-based platforms, most commonly Infinium 850k or 450k arrays (Illumina), to interrogate methylation changes on single site resolution. The 850k array examines the methylation status of 853,307 CpG sites, adding 413,745 CpG sites to the 450k (33). Six of the genome-wide studies also performed gene expression analysis (25, 26, 28, 29, 31, 32, 34).

**Table 1:** Genome-wide methylation studies under lifestyle intervention.

Lifestyle	Participants	Methods	Results	References
Comparing methylation differences between high and low responders				
Mediterranean low-carbohydrate/ fat diet with/without physical activity / **18-month** **Aim:** tested whether specific DNA methylation changes reflect individual responsiveness to lifestyle intervention	120 subjects (90% men, BMI = 30.2 ± 3.3 kg/m^2^, age = 49 ± 9 years); 10 responder (− 16% absolute weight loss) vs. 10 non-responder (+ 2.4% weight gain)	Illumina HumanMethylation 850K Bead Chips **Tissue:** blood	variation in DNA methylation of genes (DMRs) including *LRRC27, CRISP2*, and *SLFN12* between responder and non-responder;15 CpGs being negatively correlated with weight change after intervention such as *NUDT3* and *NCOR2*; baseline DNA methylation score better predicted successful weight loss than predictors such as age and BMI	(Keller et al. 2020) (23)
hypocaloric diet / **4 months** **Aim:** identify DMRs in subjects with obesity that predict the response to a weight loss dietary intervention	201 subjects with overweight (BMI: 25.0–29.9) & obesity (BMI: 30–40 kg/m^2^); 64 responder (lost 4.55 ± 0.91 kg/m^2^) & 63 non-responder (lost 1.95 ± 0.73 kg/m^2^)	Illumina HumanMethylation 850K Bead Chips **Tissue:** blood	DNA methylation analysis between responder and non-responder exhibited a DMR located at *PON3* consisting of 13 CpG sites, eleven of them significantly hypermethylated in responder.; 63 CpG sites were identified between responder and non-responder, (45 CpG sites were hypermethylated and 18 CpG sites were hypomethylated)	(Salas-Pérez et al. 2022) (24)
low-calorie diets / **6-month** **Aim:** identify novel genes that distinguish individual responses to a weight loss dietary treatment	47 subjects (WC> 94 cm males and > 80 cm females); 31 low responder (weight loss < 8%, Age = 46.5 ± 9.6, 18 male/13 female) vs. 16 high responder (weight loss > 8%, Age = 52.1 ± 9.5, 7 male/9 female)	Illumina HumanMethylation 450K Bead Chips **Tissue:** blood	analysis of both array data identified four genes: *CD44, ITPR1, MTSS1* and *FBXW5* that were differentially methylated and expressed between groups; *CD44* showed higher expression and lower DNA methylation levels in low responder than in high responder; differences in CD44 protein levels between low responder and high responder were not statistically significant, but a positive association was observed between *CD44* mRNA expression and protein levels	(Samblas et al. 2019) (25)
DNA methylation changes in response to weight loss				
Mediterranean (MED) diet enriched in polyphenols and reduced in red/processed meat (green-MED) / **18-month** **Aim:** analyse the effects of the green-MED diet on methylome and transcriptome levels	260 participants (BMI = 31.2 kg/m^2^,mean age = 51 years, 28 female / 232 male)	Illumina HumanMethylation 850K Bead Chips **Tissue:** blood	1573 DMRs were found in the green-MED compared to the MED (177) and HDG (377) diet; 1753 DEGs in the green-MED intervention compared to MED (7) and HDG (738); the highest number (6 %) of epigenetic modulating genes was transcriptionally changed in subjects participating in the green-MED intervention; *KIR3DS1* locus, is negatively associated with the polyphenol changes, but positively associated with the MRI-assessed superficial subcutaneous adipose area-, weight- and waist circumference- 18-month change	Hoffmann et al. 2023 (26)
weight loss dietary intervention / **2-years** **Aim:** investigated the relationship of DNAm levels at birthweight-blood pressure genes with long-term changes in blood pressure	672 adults with overweight or obesity (BMI = 32.7 kg/m^2^, Age = 50.9 years, 411 female / 261 male)	Illumina NovaSeq6000 by a high-resolution methyl-capture sequencing (MCC-Seq) **Tissue:** blood	DNA methylation at *LINC00319*, showed significantly different associations with 2-year changes in systolic blood pressure and diastolic blood pressure among participants assigned to low- or high-fat diets; higher regional DNA methylation at *LINC00319* was associated with greater reductions in systolic blood pressure and diastolic blood pressure	Kou et al. 2023 (27)
diabetes-specific social support program / **3-month** **Aim:** explore the association of monocyte inflammation using epigenetic, immunologic, and clinical measures	8 individuals with diabetes mellitus (BMI = 36.2 ± 5.2 kg/m^2^; 62.5% male, mean Age = 48,7)	Illumina HumanMethylation 450K Bead Chips microarray **Tissue:** blood	1,061 differentially methylated loci (DML) were identified in monocytes at baseline and 3 months; DML were enriched within genes involved in immune, metabolic, and cardiometabolic pathways; immune function showed improvement post-DM-SSP compared with baseline, characterized by attenuated interleukin 1β and IL-6 secretion from monocytes	Dye et al. 2022 (28)
VLCKD / **6 months** **Aim:** identify the changes in the obesity-related methylome that are mediated by the induced weight loss or are dependent on ketosis	21 patients with obesity (12 women, BMI= 33.0 ± 0.2 kg/m^2^, Age 47.9 ± 1.02 years)	Illumina HumanMethylation 850K Bead Chips **Tissue:** blood	after weight reduction, differences were found at 988 CpG sites; most of the encoded genes were involved in metabolic processes, protein metabolism, and muscle, organ, and skeletal system development; genes including *ZNF331*, *FGFRL1* (VLCKD-induced weight loss) and *CBFA2T3*, *C3orf38*, *JSRP1*, and *LRFN4* (VLCKD-induced ketosis)	Crujeiras et al. 2021 (29)
hypocaloric dietary intervention/ **6 weeks** **Aim:** investigate the effects of short-term hypocaloric diet-induced weight loss on DNA methylation profile in leukocytes from women with severe obesity	11 women with morbid obesity (Age = 36.9 ± 10.3 years; BMI: 58.5 ± 10.5 kg/m^2^)	Illumina HumanMethylation 450K Bead Chips microarray **Tissue:** blood	intervention changed the methylation levels at 16,064 CpG sites; These CpGs sites were related to cancer, cell cycle-related, MAPK, Rap1, and Ras signaling pathways; regardless of hypocaloric intervention, a group of 878 CpGs (related to 649 genes) remained significantly altered in obese women when compared with normal-weight women; Pathway enrichment analysis identified genes related to the cadherin and Wnt pathway, angiogenesis signaling, and p53 pathways by glucose deprivation	Nicoletti et al. 2020 (30)
weight loss intervention / **1 year** **Aim:** examine whether weight loss and acquired obesity produce reciprocal profiles	19 healthy obese participants (mean BMI = 34.6 kg/m^-2^, 7 males/12 females, Age = 35.2±1.8)	Illumina HumanMethylation 450K Bead Chips **Tissue:** SAT	7 genes (*UCHL1*, *BAG3*, *TNMD*, *LEP*, *BHMT2*, *EPDR1* and *OSTM1*) downregulated during both short- and long-term weight loss	Bollepalli et al. 2018 (31)
MBC2 healthy diet/**9-month** **Aim:** examine the impact of the MBC2 and activity intervention on patterns of epigenome-wide DNA methylation.	340 adults with non-optimal levels of health behaviors (Age = 18-65, primarily women, BMI= 34.5-37.3 kg/m^2^)	Illumina HumanMethylation 850K Bead Chips **Tissue:** blood	no differentially methylated regions at baseline between the control versus intervention groups; 3 versus 9 months: 154 and 298 differentially methylated regions between controls compared to sequential and simultaneous groups; overlap between 3 and 9 months, including the GDP-L-fucose biosynthesis I, methylmalonyl metabolism, and estrogen-mediated cell cycle regulation pathways	Hibler et al. 2019 (32)

This table summarize our genome-wide methylation studies under lifestyle intervention and includes comparing of methylation differences between high and low responders and DNA methylation changes in response to weight loss. BMI, body mass index; DMR, differentially methylated regions; LRRC27, leucine rich repeat containing 27; CRISP2, Cysteine rich secretory protein 2; SLFN12, Schlafen family member 12; NUDT3, nudix hydrolase 3; NCOR2, nuclear receptor corepressor 2; PON3, Paraoxonase 3; ITPR1, inositol 1,4,5-trisphosphate receptor type 1; MTSS1, Metastasis Suppressor 1; FBXW5, F-Box and WD repeat domain containing 5; DML, differentially methylated loci; DM-SSP, diabetes mellitus -specific social support program; VLCKD, very-low calorie ketogenic diet; ZNF331, zinc finger protein 331; FGFRL1, fibroblast growth factor receptor like 1; CBFA2T3, CBFA2/RUNX1 partner transcriptional co-repressor 3; C3orf38, chromosome 3 open reading frame 38; JSRP1, junctional sarcoplasmic reticulum protein 1; LRFN4, leucine rich repeat and fibronectin type III domain containing 4; MAPK, mitogen-activated protein-kinase; Ras, Rat sarcoma; Rap1, Ras-related protein 1; Wnt, wingless-type; UCHL1, ubiquitin C-terminal hydrolase L1; BAG3, BAG cochaperone 3; TNMD, tenomodulin; LEP, leptin; BHMT2, betaine–homocysteine S-methyltransferase 2; EPDR1, ependymin related 1; OSTM1, osteoclastogenesis associated transmembrane protein 1; MBC2, Make Better Choices 2; GDP, guanosin diphosphate.

### DNA methylation associated with weight loss

Three of the reviewed genome-wide intervention studies aimed to identify DNA methylation biomarkers for response to weight loss. This was accomplished by comparing baseline and endpoint methylation differences between high and low responders to an individual intervention. The individual response to the weight loss intervention was defined differently within the three studies. In the study published by Keller et al., the responder group lost at least 10 % of their initial body weight, whereas in the study by Samblas et al., subjects were defined as high responders already with a weight reduction of > 8 % (23, 25). In the third study, the change in BMI was used to classify responders (lost 4.55 ± 0.91 BMI units (kg/m^2^)) and non-responders (lost 1.95 ± 0.73 kg/m^2^) rather than mean weight loss (24). Two studies intervened with different low-calorie diets (24, 25), while the other study used a Mediterranean diet combined with physical activity (23). Study intervention times ranged from four months to 18 months and the number of participants varied from 47 to 201 (**Table 1**). Among them two studies identified DMRs (23, 24) whereas the third only reported DMPs (25). The identified DMRs and DMPs from the three studies retained their significance even after correction for multiple testing. Among the identified DMRs five were significantly hypermethylated (*CRISP2, SLC6A12, SLFN12, AURKC, PON3*) and four significantly hypomethylated (*LRRC27, RNF39, LINC00539, NTSR1*) in responders compared to non-responders (23, 24). DNA methylation analysis by Salas-Pérez et al., not only identified one DMR at *PON3*, but also 63 CpG sites that were differentially methylated between these two groups (24). The third study revealed four genes, *CD44, ITPR1*, *MTSS1* and *FBXW5* by overlapping identified DMPs with differentially regulated transcripts (25). We were able to find other overlaps in genes between the three studies, but these did not withstand multiple testing. Although no overlap in differentially methylated genes could be observed between the three newly published studies in regard to weight loss response, such studies indicate that methylation differences at multiple genomic sites could serve as prognostic biomarkers to predict successful weight loss therapy. However, validation is still essential in order to delineate the real potential of DNA methylation pattern in predicting individual’s response to a specific lifestyle therapy.

The remaining seven studies measured DNA methylation changes in response to weight loss. Most of the studies intervened with a low-calorie diet, and some studies also combined this change in diet with physical activity (31, 32). One study even compared three different dietary interventions as addition to physical activity and thereby investigated the beneficial effect of dietary polyphenols (26). Personalised weight loss programs have also been used, including T2D-specific social support programs, aiming to reduce risk factors associated with diabetes complications (28). Study intervention times ranged from six weeks to two years and the number of participants varied from eight to 672 (**Table 1**). Most of these studies used Benjamini Hochberg to correct their results for multiple testing with a false discovery rate (FDR) < 0.05 to detect changes in DMRs and DMPs. All studies examined DNA methylation in blood samples except for the study by Bollepalli et al., which examined subcutaneous adipose tissue (SAT) (31). Numerous DMRs with different functions have been identified. Those DMRs were annotated to genes mainly involved in e.g. immune, metabolic, and cardiometabolic signalling pathways, protein metabolism, and the development of muscles, organs, and skeletal systems. In addition, the study by Crujeiras et al. provided evidence of obesity-related methylome remodeling after dietary interventions, finding similar levels of methylation in treated obese patients as in normal-weight individuals. In fact, in the same study the most representative genes *ZNF533* and *FGFRL1*, which were differentially methylated after dietary weight loss treatment, were previously identified as an epigenetic signature of adipose tissue associated with obesity, which is reflected in blood leukocytes (22). The study by Hoffmann et al. demonstrated many DMRs induced by the three different dietary interventions and further showed that a Mediterranean diet enriched in plant-based polyphenols has the highest capacity to regulate individual’s blood epigenome (26). This was the so far largest long-term RCT, analyzing lifestyle effects on human DNA methylation pattern. Thus, we performed an intersection analysis comparing all identified unique genes from the other six genome-wide approaches that showed statistically significant (FDR < 0.05) weight loss-related changes in DNA methylation with the results of Hoffmann et al. (26). The majority of the 2097 genes identified in the seven studies focusing on genome-wide methylation changes were unique (N=2051), however 47 genes were reported by at least two intervention studies and one gene, the *Mitotic Arrest Deficient 1 Like 1* (*MAD1L1*) was even identified by three independent studies (26, 28, 29). This gene is part of the mitotic spindle-assembly checkpoint and thus plays a major role for cell cycle control with potential involvement in cancer development (35). Furthermore, other known candidate genes for obesity and T2D such as *TCF7L2* locus, which was previously reported by Aronica et al. (8), were now validated again by two other lifestyle studies (26, 28).

While further intersecting those unique genes with the studies associating DNA methylation with weight loss response, we found 518 genes reported by both study types. Among them five genes (*BCAS4*, *MYH15*, *SH3PXD2A*, *VIPR2* and *WDPCP*) were even reported by two weight loss response studies. Among them the *WD repeat containing planar cell polarity effector (WDPCP)* gene (24, 31) was identified by Salas-Perez et al. reporting one DMP in *WDPCP*, which was hypomethylated (~5%) in responders compared to non-responders of the weight loss intervention (24). This goes in line with the results of Bollepalli et al., who found an upregulation of the *WDPCP* gene expression in the heavier co-twins (31).

### Epigenetic regulation of physiological candidates

Our search revealed only nine new candidate gene studies in the last five years. All studies compared DNA methylation at baseline and after finishing an individual lifestyle intervention for one or several candidate genes. The studies employed different methods including pyrosequencing, although array-based platforms have been dominating in the last years. Therefore, it is not surprising that candidate gene analyses were frequently based on previously generated and available genome-wide datasets. The candidate gene analyses were performed in interventional studies based on diet or a combination of diet and physical activity. Only the study by Willmer et al. intervened solely with physical activity (36). The candidate gene studies also varied greatly in the number of participants from 18 to 811 subjects, and the duration of the intervention from three weeks to two years (**Table 2**).

**Table 2:** Candidate-gene methylation studies under lifestyle intervention.

Lifestyle intervention	Participants	Methods	Gene(s)	Results	Reference
weight loss diet interventions varying in macronutrient components/**2-years** **Aim:** investigate the association of DNA methylation at the *CPT1A* gene with reductions in triglycerides and triglyceride-rich lipoproteins (TRLs) in response to weight loss diet interventions	528 participants (BMI = 32,5 kg/m^2^, Age = 52 years, 305 female/223 male)	IlluminaNovaSeq6000 platform by a high-resolution methyl-capture sequencing (MCC-Seq)	*CPT1A*	Dietary fat intake significantly modified the association between baseline DNA methylation at *CPT1A* and 2-year changes in total plasma triglycerides, independent of concurrent weight loss; with low-fat diet, a higher regional DNAm level at CPT1A was associated with a greater reduction in total plasma triglycerides at 2 years compared to a high-fat diet	Li et al. 2023 (37)
energy-reduced diets/**2-year** **Aim:** examine the impact of the *NFATC2IP* rs11150675 genotype on adiposity changes	692 overweight and obese people (BMI = 25-40 kg/m^2^, mean Age = 51.4 years, 61.1% females)	OpenArray SNP Genotyping System & Illumina HumanMethylation 450K Bead Chips **Tissue:** blood	*NFATC2IP*	dietary fat intake significantly modified the effect of the genetic, epigenetic and transcriptional variations at the *NFATC2IP* locus of weight change; *NFATC2IP* methylation mediated 52.8% of its genotypic effect in response to a high-fat diet rather than a low-fat diet	Sun et al. 2018 (38)
weight loss diet intervention/**2-years** **Aim:** investigate whether baseline blood DNA methylation levels in *TXNIP* can be associated with glycemic characteristics and their changes in response to weight loss interventions	639 adult participants with overweight or obesity (BMI= 25-40 kg/m^2^,mean age= 50.1-52,2)	IlluminaNovaSeq6000 platform by a high-resolution methyl-capture sequencing (MCC-Seq) immunoassay with chemiluminescent detection on an Illumina analyzer **Tissue:** blood	*TXNIP*	higher regional DNA methylation at *TXNIP* was significantly correlated with lower fasting glucose, HbA1c, and HOMA-IR at baseline; dietary protein intakes significantly modified the relation between regional DNA-methylation level at *TXNIP* and changes in insulin and HOMA-IR at 6 months	Li et al. 2022 (39)
supervised aerobic & resistance training/**12-weeks** **Aim:** investigate ASAT and GSAT DNA methylation of *FKBP5* in response to an exercise intervention	19 African women with obesity (BMI = 34.9 kg/m^2^, mean age = 22) 12 controls continued their usual behaviour (BMI = 33.0 kg/m^2^, mean age = 24)	Pyrosequencing, SNP & gene expression analyses with real-time PCR **Tissue:** GSAT & ASAT	*FKBP5*	Exercise training induced *FKBP5* hypermethylation at two CpG dinucleotides within intron 7; CC allele carriers displayed improved cardiorespiratory fitness, insulin sensitivity, gynoid fat mass, and waist circumference	Willmer et al. 2022 (36)
lifestyle intervention (Care Call programme)/**6 months** **Aim:** investigated whether a lifestyle intervention could influence expression and DNA methylation of diabetes-related genes	20 participants with impaired glucose regulation (10 females/10 males, Age = 18–80)	Pyrosequencing **Tissue:** blood & adipose tissue	*CAV1*	intervention resulted in opposite direction changes in fat tissue and blood for *CAV1* expression and DNA methylation and these changes were correlated between tissues	Fachim et al. 2020 (40)
BWRP/**3-week** **Aim:** evaluate the DNA methylation status of seven *clock* genes	45 obese adolescents (BMI = 37.5 kg/m^2^, 28 female/17male, Age = 15.8 ± 1.4)	Pyrosequencing **Tissue:** blood	*CLOCK, PER1-3 & CRY1-2*	BWRP changes in the methylation levels of *CLOCK, CRY2* and *PER2* genes; hypermethylation of *CLOCK* and *PER3* genes in males and in subjects with metabolic syndrome	Rigamonti et al. 2022 (41)
weight loss program intervention/**6-month** **Aim:** quantify *FTO* whole blood DNA methylation & investigate the relationship between body composition, exercise capacity & blood parameters	18 female participants (BMI: 33.5 ± 6.2 kg/m^2^, mean age, 50.6 ±12.1 years)	Pyrosequencing **Tissue:** blood	*FTO*	Methylation rate was significantly decreased in the normal treatment group in CpG1; treatment group containing resistance training CpG3 was increased	Nishida et al. 2020 (42)
Mediterranean low-carbohydrate/fat diet with/without PA/**18-month** **Aim**: examine the effect of lifestyle interventions on DNA-methylation of nonalcoholic fatty-liver disease related genes	120 participants from the CENTRAL RCT, (92% men; BMI = 30.2 kg/m^2^, age = 49 ± 9 years)	Illumina HumanMethylation 850K Bead Chips; Single-nucleotide polymorphisms genotyped by TaqMan assays **Tissue:** blood	*AC074286.1, CRACR2A, A2MP1, FARP1*	Baseline-IHF% was inversely correlated with DNA-methylation within *AC074286.1, CRACR2A, A2MP1, FARP1*; differential DNA-methylation patterns were observed between diets at *A2MP1* and between PA groups within AC074286.1, CRACR2A, and FARP1 CpGs	Yaskolka Meir et al. 2021 (23)
VLCKD, HCD or BS/**4–6 months** **Aim:** evaluate the methylation levels of ACE2 gene, the main entry receptor of SARS-CoV-2, in different depots of AT (subcutaneous and visceral) and PBMCs	45 obese patients (23 female/22 male) compared with non-obese patients (9 female/9 men)	Illumina HumanMethylation 450K Bead Chips **Tissue:** SAT, VAT, PBMCs	*ACE2*	VAT from patients with obesity showed higher ACE2 methylation levels, mirrored in PBMCs but not in SAT; observed obesity-associated methylation of ACE2 was reversed after VLCKD and HCD but not after BS; observed DNA methylation pattern was inversely correlated with ACE2 expression	Izquierdo et al. 2022 (34)

This table summarize our candidate-gene methylation studies under lifestyle intervention. SNP, Single nucleotide polymorphism; NFATC2IP, Nuclear factor of activated T cells 2 interacting protein; ASAT, abdominal subcutaneous; GSAT, gluteal subcutaneous adipose tissue; PCR, polymerase chain reaction; FKBP5, FKBP Prolyl Isomerase 5; CAV1, Caveolin 1; BWRP, multidisciplinary body weight reduction program; CLOCK, Circadian locomoter output cycles protein kaput; CRY2, Cryptochrome circadian regulator 2; PER2/3, Period circadian regulator 2/3; FTO, Fat mass and obesity-related; IHF, intrahepatic fat; PA, physical activity; CRACR2A, Calcium release activated channel regulator 2A; A2MP1, Alpha-2-macroglobulin pseudogene 1; FARP1, FERM, ARH/RhoGEF and pleckstrin domain protein 1; HCD, a balanced hypocaloric diet; VAT, visceral adipose tissue; SAT, subcutaneous adipose tissue; BS, bariatric surgery; ACE2, Angiotensin converting enzyme 2.

These studies analysed the methylation status of several genes related to immune response (*NFATC2IP* & *FKBP5*) (36, 38), mitochondrial function (*CPT1A*) (37) diabetes and ageing (*TXNIP* & *CAV1*) (39, 40), circadian rhythm (*CLOCK, CRY2 & PER2*) (41) as well as obesity related genes (e.g. *FTO*) (42). These genes were chosen based on previous studies because they were, among other things, associated with metabolic control, glucose homeostasis or obesity.

Because most studies examined different genes, reproducibility between the different studies is lacking, although genes such as *FTO, PER2* and *CLOCK* were already reviewed by Aronica et al. (43, 44). The *Fat Mass and Obesity-associated* (*FTO*) gene was the first gene to be associated with body fat, obesity and BMI (45, 46). A study from 2017 reported that hypomethylation of the *FTO* non-promoter region is an early marker of T2D (47) and another study showed that hypomethylation induces overeating, fat accumulation, and obesity (48). The study we found suggests that resistance training increased DNA methylation in the *FTO* 5’UTR region due to weight loss and thereby may repress *FTO* mRNA expression (42). Thus, the epigenetic regulation of *FTO* might also play a role in obesity development, but further replication studies with larger sample sizes as well as functional analyses are warranted.

Chronobiological misalignment and disrupted physiological rhythms has been repeatedly linked to obesity (49, 50) and genes previously shown to be associated with the regulation of circadian rhythm such as *CLOCK* are likely to play a crucial role (51). A study by Milagro et al. from 2012 showed that the baseline methylation levels of *CLOCK* and *PER2* are correlated with the magnitude of weight loss (43). A recent study by Rigamonti et al. showed that a short-term body weight reduction program induced a significant *CLOCK* hypermethylation together with a significant *PER2* hypomethylation, suggesting that the body weight reduction program might result in beneficial cardiometabolic effects as well as in epigenetic remodelling of specific *CLOCK* genes. However, due to the experimental design, this study could not disclose whether epigenetic changes in *CLOCK* genes are cause or consequence of global cardiometabolic improvements (41).

### Weight loss driven improvement of methylation age

With increasing age, the risk of mental and physical impairment and the development of diseases such as cancer, neurodegeneration, T2D and cardiovascular disease (52, 53) is also rising. Epigenetic changes can be induced by multiple factors such as genetics (54), environment (55) and lifestyle (56). In addition to molecular and cellular characteristics, epigenetic changes are affected by ageing but can also influence the ageing process itself (57). Out of more than 20 million methylation sites in the human genome, there are several thousand where the methylation levels correlate closely with age (58). Methylation age acceleration, defined as the difference between biological age (age of cells and tissue based on physiological evidence) and chronological age (actual age in years), is used to study the links between epigenetic ageing and disease development (59) and it has been implicated not only in obesity (60), but also in physical fitness (61) and stress (62). Cross-sectional studies have already provided evidence of a beneficial effect of a healthy lifestyle on several biological indicators of ageing such as epigenetic clock, telomere length and transcriptomic age (63, 64). However, little is known about the interaction between epigenetic ageing and lifestyle factors, including diet, alcohol abuse and physical activity. Therefore, longitudinal and interventional studies are needed to address and accurately quantify the benefit of lifestyle improvements on ageing. Thus, intervention studies can help to identify factors that have a positive influence on epigenetic age and thus contribute to improving general health.

So far, two intervention studies that examined changes in epigenetic age through weight loss and a healthier lifestyle in overweight and obese patients have been reported since 2018 (**Table 3**). One study linked physical functioning to biological age in older adults (65) whereas the other showed that lifestyle-based weight loss interventions can indeed reduce DNA methylation age (mAge) (66). Furthermore, Yaskolka Meir et al. demonstrated associations between mAge acceleration and parameters of body fat distribution and glycaemic control (66). Thus, DNA methylation-based biological measures of ageing might allow to stratify the individual health status and to predict risk of premature cardiometabolic diseases (66). It is thereby possible to investigate positive effects of lifestyle improvements on age-related epigenetic mechanisms (67). Yet, the results could be influenced by other important factors, and so, it is still elusive whether weight loss was the main reason for the positive biological effect (66). However, both studies were able to show that the epigenetic age is strongly correlated with health and could therefore be considered as an important biomarker for outcome predictions in human intervention studies.

**Table 3:** Weight loss driven improvement of methylation age.

Study	Participants	Methods	Results	References
weight loss intervention/**18-month** **Aim:** evaluated the role of multiple factors on the deviation of mAge from chronological age	120 CENTRAL participants with abdominal obesity or dyslipidemia (BMI = 30.2 ± 3.3, 10 female/110 male, Age = 48.6 ±9.3) **Tissue:** blood	Illumina HumanMethylation 850K BeadChips	with abdominal obesity or dyslipidemia, mAge was higher than the chronological age; weight loss lifestyle intervention attenuated the mAging of the men by 7.1 months	Yaskolka Meir et al. 2021 (66)
Weight loss intervention/**12 weeks** **Aim:** examined the association between changes in physical function and DNA-methylation-based biological age at baseline and 12 weeks.	16 older adults with obesity (BMI= 36.2 ± 7.0, Age = 73.50 ± 5.72, 14 female/2 male) **Tissue:** blood	Illumina HumanMethylation 850K BeadChips	Participants mean weight loss was 4.6 kg and DNA methylation age decreased by 0.8; decreased methylation age was associated with significantly increased gait speed	Peterson et al. 2021 (65)

This table summarizes the studies, which deal with the improvement of the methylation age through weight loss. mAge, methylation age.

### Weight loss driven methylation remodelling during pregnancy

Maternal overweight and obesity is associated with the risk of overweight and obesity in children (68). Underlying mechanisms which contribute to the increased susceptibility for overweight and obesity and associated health consequences in children are not yet fully understood (69–72). Furthermore, overweight and obesity of the mother, weight gain during pregnancy, prenatal nutrition and physical activity have a direct impact on the health of the foetus (68). Those intrauterine disturbances can lead to epigenetic remodelling (6, 73) and further influence health outcomes in the offspring (74). In line, previous studies have shown the involvement of dietary micronutrients, after birth and during early life, in altering gene expression and thereby influencing health and disease later in life (75, 76). Moreover, low birth weight is a risk factor for developing non-insulin dependent diabetes mellitus (NIDDM) and cardiovascular disease (CVD) later in life (77, 78) and the delivery (caesarean section or vaginal delivery) plays an important role in the formation of the microbiome (79–82). The development of the child’s microbiome is determined by proper nutrition and the transition from breastfeeding to solid food (79). The microbiome normally returns to a state of equilibrium following stress such as a change in diet, short-term antibiotic treatment, or acute invasion by a pathogenic bacterium (83). However, short-term modulations of the gut microbiome can disrupt normal metabolite production (84) and this can lead to changes in host gene expression, which in turn could trigger longer-lasting effects in the host. For example, germ-free mice, a method used to determine whether the microbiome plays a causal role in regulating gene expression, showed lower genome-wide DNA methylation in colonic tissue compared to control animals (85). However, global DNA methylation increased dramatically in the germ-free mice after a fecal transplant.

Our systematic review highlighted five studies in the last five years which examined lifestyle interventions in pregnant women with obesity or overweight. They demonstrated clear associations between DNA methylation changes and health benefits of the mother and the child, such as a protective effect against the development of gestational diabetes (86), a beneficial effect on improving glycaemic control (87) and a reduced risk of the new born for obesity and obesity-related disorders later in life (88). These five studies reported genome-wide analyses following interventions with diet or a combination of diet and physical activity during pregnancy. Numerous genes were identified showing significantly altered DNA methylation after diet or physical activity interventions, such as *DISC1*, *GBX2*, *HERC2*, *HUWE1* (89), *LGR6* (90), *RNF214*, *PCSK7*, *SYN3*, *JARID2*, *POLR2C* (91). Nevertheless, one study showed no effect of the intervention neither on DNA methylation nor on the maternal BMI in early pregnancy (92). Of note, two of the identified genes, *JARID2* and *LGR2* have been reported in the above mentioned studies comparing responders and non-responders (23–25). It needs to be noted that all studies measured DNA methylation in cord blood samples which might not be a reliable indicator of the DNA methylation in the infant. In addition, umbilical cord blood contains different cell types, which may be present in different proportions in different samples, possibly distorting the effects of interest (93). Therefore, all analyses are adjusted for estimated cell type proportions and the true cell type proportions in the samples remain unknown. Nevertheless, since drawing cord blood is non-invasive and blood can be obtained in large quantities, it is often used for DNA methylation studies in pregnant women (94). Newly reviewed studies, as well as previous work, suggest that a healthy lifestyle during pregnancy is immensely important to reduce the risk of childhood obesity and the serious diseases associated with it, though the results are not consistent and challenging to compare as each study identified various differently methylated sites. Often these are single CpG sites located in different regions of the genome and with an unknown association to overweight, obesity or growth (95, 96). Furthermore, explicit attempts to replicate the findings from other studies have so far not been successful (96–98). Given the scarceness and the heterogeneous character of the available studies, there is an urgent need to perform large-scale analyses elucidating epigenetic foetal markers that are influenced by parental lifestyle (99).

## Discussion

We conducted a systematic literature review on DNA methylation analysis in individuals with overweight or obesity who underwent a weight loss intervention program driven by lifestyle modifications. In addition, we also looked for studies analysing DNA methylation pattern as a marker for weight loss response. We focused on DNA methylation only since it is by far the most extensively studied epigenetic modification and can be used to quantify allele specific epigenetic changes on a single nucleotide resolution (8). In contrast, there are no human intervention studies examining histone modifications and only a few studies in mice which characterised histone modifications by lifestyle interventions (100, 101). However, when it comes to DNA methylation, it must be noted that many of the genome-wide approaches are still array based and thus only analyse around 1.5 - 2.8 % of all CpG sites in the human genome. Although sequencing-based methods with higher coverage have recently became more accessible and cost-effective, a large part of the human epigenome remains unexplored especially in regard to lifestyle interventions and obesity. Numerous new DNA methylation profiling methods such as reduced representation bisulfite sequencing, methylation capture bisulfite sequencing and advanced microarrays have been established over the past decades, expanding our understanding of DNA methylation in diseases such as obesity. Consequently, advances in DNA methylation have shifted the challenge from big data generation to data analysis (102).

The majority of epigenetic studies related to obesity are cross-sectional and lack measurements and potential adjustments for relevant lifestyle factors. Therefore, we exclusively chose human intervention studies in our review, as these overcome some biases, such as inferring false associations or those not driven by lifestyle factors. As mentioned above, we also excluded surgical intervention studies, since they represent major metabolic remodelling, including shortage of supply and are thus less comparable to standard lifestyle therapies based on diet and physical training (30, 103). Furthermore, patients eligible for a surgical intervention are characterised with higher degree of obesity, which is linked to a more frequent prescription of drugs targeting the associated comorbidities. In addition, also other factors affecting DNA methylation levels in post-surgical care, such as the prescription of vitamin/mineral supplements to prevent nutritional deficiencies, would compromise comparisons between surgical and dietary interventions (30, 103). We found 19 human intervention studies in our literature search which were published during the last five years, of which nine were candidate gene based and ten were genome-wide approaches. Among the genome-wide studies, three studies compared responders and non-responders to an individual lifestyle intervention and seven the effect of the intervention on DNA methylation changes.

### Genome-wide DNA methylation changes

Compared with candidate gene approaches, the number of genome-wide analyses has increased greatly in the last five years (**Figure 1**), necessitating this updated discussion and bringing hypothesis-free studies more into focus (8).

Among them three studies showed baseline methylation levels at multiple genomic sites to be associated with individuals’ propensity to lose weight on specific lifestyle treatments. Although no overlap in response related methylation marks could be found between the three interventions, partially driven by differences in type of intervention, its duration, sample size and analytical approaches, they clearly suggest using blood DNA methylation marks as prognostic markers. Among the identified genes *CD44* represents an interesting candidate since its hepatic mRNA expression was previously described to be enriched in morbidly obese individuals (104) and is associated with the development of adipose tissue inflammation and insulin resistance (105). Thus, *CD44* renders a biomarker for insulin resistance and a possible therapeutic target for T2D treatment (106). In line with a previous work, showing that CD44 methylation can contribute to weight loss prediction (107) a recent work from the same lab showed that *CD44* had higher expression and lower levels of DNA methylation in low responders compared to high responders through a low-calorie diet intervention. *CD44* is involved in amplifying the inflammatory processes in obese individuals, and increased expression of *CD44* prior to a calorie-restricted diet intervention may impair its effectiveness in terms of a successful weight loss (25). In addition to *CD44*, genes such as *ATP10A, AQP9, and HIPK3* have previously been discussed by Aronica et al. in regard to weight loss response and were also identified in the latest studies comparing responders and non-responders to an individual calorie restriction intervention (23, 107, 108). However, they did not withstand corrections for multiple testing, as has also been shown for a number of genes in the three responder/non-responder studies (including *RNF39, VIPR2, IGHMBP2, SH3PXD2A, WDPCP, VWDE, SYNJ2, VWDE, BCAS4, MYH15, PCDHGA4, KCNG2, FRMD4A, STK32C* and *LGR6*). Although these genes may still be of considerable interest in the respective research field, unless validated in further studies, they should be seen with caution.

By intersecting the results of all studies analysing genome-wide methylation changes based on lifestyle interventions we identified 47 genes which were affected in two independent studies and even one gene which was reported by three studies – *MAD1L1*. Although the role of this cell cycle and tumor suppression relevant gene for obesity was not yet investigated, one recent study showed differently blood methylation of *MAD1L1* in infants of obese mothers (109). Furthermore, DNA methylation of this genes in visceral adipose tissue may contribute to the discrimination of patients with and without colorectal cancer (110). In addition, two new studies reported DNA methylation changes on *TCF7L2* which was previously reported by Aronica et al., and is a well-known candidate gene for T2D as identified by GWAS (111). The genetic risk variant was reported to alter mRNA expression levels and decrease insulin secretion in the pancreatic ß-cells (112). In line, the methylation status of *TCF7L2* is altered in T2D islets (110) and was previously shown to be modified in adipose tissue under physical activity training (113).

Finally, we aligned those 47 candidates with those genes being potentially involved in weight loss response. Thereby, we could identify 518 genes. Among the five genes which were reported several times among all the studies was *WDPCP*, which is shown to be involved in ciliogenesis and collective cell movement during embryogenesis (114). To date, there are two human intervention studies, which described altered DNA methylation or gene expression in *WDPCP* (24, 31). The study by Salas-Perez et al. identified one DMP in the *WDPCP* body, which was hypomethylated (~5%) in responders compared to non-responders of the intervention. In line with this, Bollipalli et al. found a downregulation of the *WDPCP* mRNA expression in SAT of patients who successfully lost weight one year after intervention, but no corresponding change in DNA methylation for *WDPCP* was found in this study (31). However, Keller et al. also found a change in DNA methylation of *WDPCP*, but this did not survive multiple testing (23). Both studies included healthy participants with overweight and intervened with a hypocaloric diet. However, the study by Bollepalli et al. additionally included physical activity and the duration of the intervention varied from four months to one year. In contrast, the gene was not previously reported by Aronica et al.

### Candidate gene methylation changes

In comparison to the genome-wide studies, no overlaps could be found between the candidate-gene studies. However, there are some overlaps with previous studies reported by Aronica et al. This includes the genes *CLOCK, PER2* and *FTO*. Furthermore, overlaps between the genes *TXNIP* (39), *CAV1* (40), *FTO* (42) and CRY (41) were recently identified in regard to weight loss response (23), as CpG sites among those genes were included in a weight loss prediction model.

Nevertheless, the lack of reproducible results even within comparable diet regimes can be explained by differences in studies regarding factors like gender distributions, ethnicity, BMI, sample size or duration of intervention. For example, the number of participants varied from 8 to 672 in the genome-wide studies and between 18 and 811 in candidate gene approaches. Furthermore, presence of comorbidities has been differently handled and reported, and specific medications were poorly addressed within downstream analyses.

However, weight loss is a dynamic process, and the underlying physiological and potential epigenetic modifications are under permanent change. Thus, the duration of the intervention plays a major role for the results. Within the here reviewed publications intervention length ranged from two weeks to two years. Due to the large differences, DNA methylation was assessed at different stages of metabolic remodelling. This was further supported by the wide range of weight loss reported in the different studies. Furthermore, weight loss success was defined either by actual weight loss in kg, by reduction in BMI or by specific health improvements. This makes comparing weight loss success between the studies highly challenging (if not impossible) and not allowing to conduct a comprehensive meta-analysis.

### Analysed tissue

Whole blood is the most commonly used biological material in genetic and epigenetic studies as it is easy to draw and often the only available source. Studies included in this review also predominantly used blood samples. However, blood is a mixture of different cell types with different methylation patterns. Deconvolution techniques, such as constrained projection/quadratic programming (CP/QP) (19) have been further developed in recent years with the goal to improve the accuracy of the cell composition estimates and to overcome potential technical differences between the platforms (115, 116). This has been exemplarily shown in the study by Salas et al, where the available reference library for the deconvolution of blood cell fractions using the EPIC array was expanded (14). Although statistically correction for cell type composition appears essential for this kind of data (117, 118), this was only done in four of 14 studies. Nevertheless, blood DNA methylation could serve as a biomarker for weight-change despite it may mirror target tissue methylation changes only partly, which clearly warrants similar analyses in metabolic relevant target tissues such as adipose tissue. Although Aronica et al. reviewed two interventional studies in AT, since 2017 only a few studies used AT to examine changes in DNA methylation associated with weight loss. Some of these studies were able to show that DNA methylation in adipose tissue changed significantly after diet and exercise intervention (8, 31, 34, 113, 119). However, adipose tissue also consists of several cell types and only 20-40 % of these are adipocytes. The rest are fibroblasts, preadipocytes, stem cells and immune cells and during weight loss, this cell composition changes, which may also affect DNA methylation (120). In summary, further studies aiming to measure the capacity of lifestyle changes on epigenetic remodelling in AT are required.

### DNA methylation age

**Figure 2:**
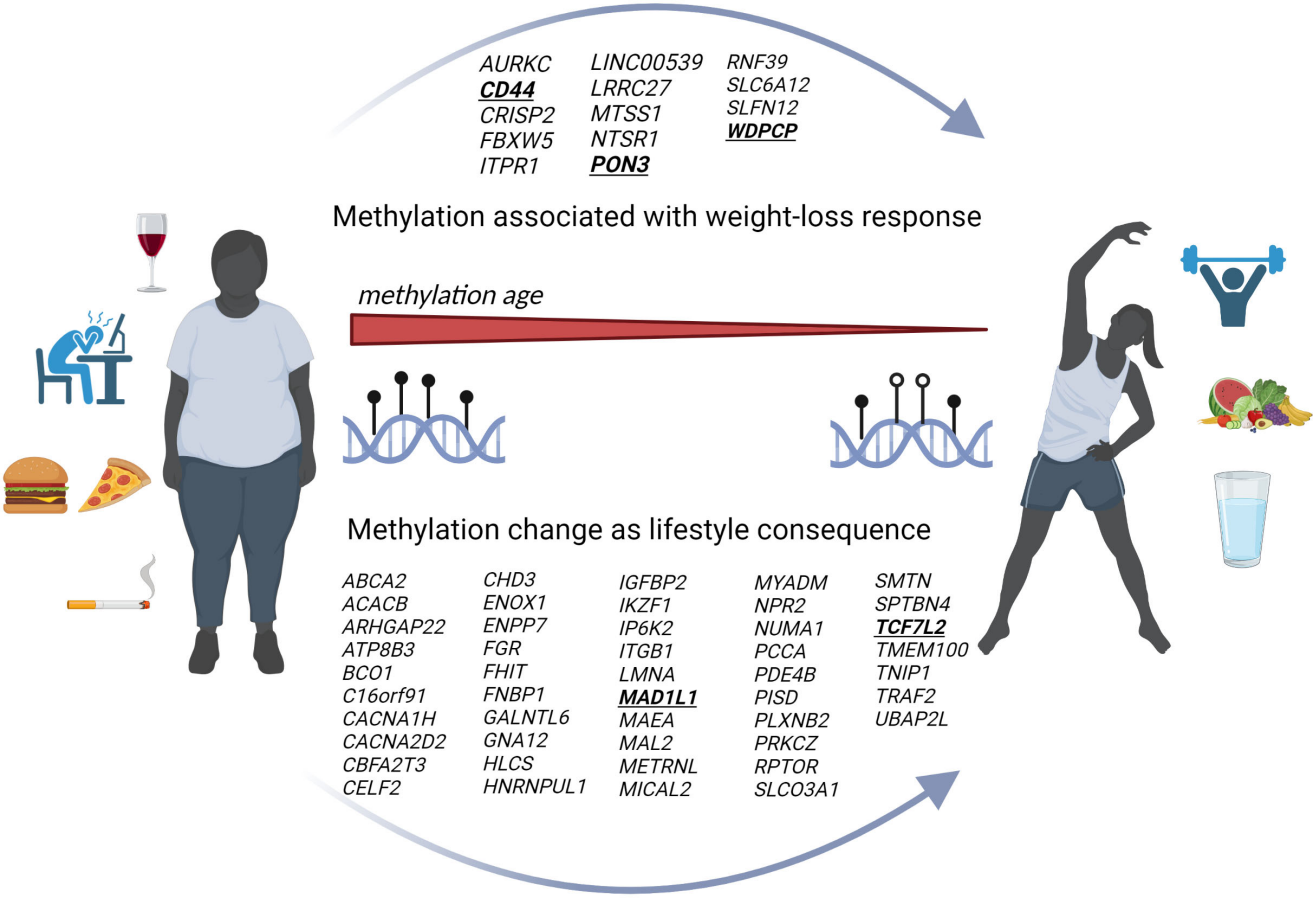
Genome-wide DNA methylation and lifestyle mediated weight loss. Scheme summarizes our results based on genome-wide DNA methylation during the previous five years. The figure indicates a reduced mAge which is associated with an improved lifestyle and weight loss. The upper arrow within the figure represents newly identified genes being associated with a weight loss success whereas the lower arrow shows all at least twice reported genes with changes on the methylome based on lifestyle modifications and weight loss. (Created with Biorender.com).

DNA methylation age, a newly established epigenetic measure, and thus not previously discussed, has received increasing attention in the last five years. Although DNA methylation is considered rather a stable epigenetic mark, the genome gains and loses methylation stochastically, whereas ageing seems to be associated with this epigenetic remodelling. Recent studies showed that higher diet quality is inversely associated with methylation-based measures of biological age and that a healthier lifestyle may play a major role in reducing biological age (121, 122). In line with this, weight loss interventions seem to reduce methylation age and the mAge acceleration which correspond to an improved metabolic and physical phenotype (**Figure 2**) (65, 66).

### Smoking as important lifestyle contributor

Although known to be strongly associated with blood methylome profiles (123–132), smoking is another important cofactor that is mostly underreported in epigenetic studies related to obesity (133, 134). The well-established association of DNA methylation levels in *AHRR* (*aryl hydrocarbon repressor repressor*) and *F2RL3* (*coagulation factor II receptor‐like 3*) genes with smoking (126, 130–132) appears particularly interesting in this context, since these two candidates were also identified in a study comparing blood methylome of subjects living a very healthy vs. a very unhealthy lifestyle based on a combined score derived from diet, physical activity, smoking and alcohol consumption (122). This further supports smoking to be a prominent lifestyle factor, potentially masking smaller effects derived from other components such as diet or physical activity (**Figure 2**). Unfortunately, most of the intervention studies have ignored the impact of smoking on DNA methylation so far, which is a clear limitation and could partially explain the lack of reproducibility in reported studies.

### In-utero reprogramming

It is well-acknowledged that epigenetic changes could be passed onto the following generation (6, 89–91, 135) (**Figure 2**). To date, there is only limited research proving that transgenerational epigenetic inheritance exists in humans (6) and, moreover, explicit attempts to replicate the results of other studies have so far failed (96–98). The fact that epigenetic changes are more dynamic and reversible poses another challenge. However, further studies are needed to develop strategies for preventing obese phenotypes in the offspring, trying to control epigenetic (re)programming during early pregnancy and breastfeeding (136, 137). Early pregnancy appears to be the most vulnerable and important phase and, interestingly, cord blood CpG site methylation of the offspring seems to be more frequently altered in underweight (N=1621) than in obese mothers (N=28) (95). According to the study by Sharp et al., paternal BMI appeared to be of less important, then weight gain during pregnancy (95). However, we identified five genome-wide analyses that intervened with diet or a combination of diet and physical activity during pregnancy. Various genes demonstrated significantly altered DNA methylation after diet or physical activity interventions, such as *DISC1*, *GBX2*, *HERC2*, *HUWE1* (89), *LGR6* (90), *SYN3*, *JARID2, POLR2C* (91). Since *JARID2* and *LGR2* were also found in intervention studies aiming to identify DNA methylation biomarkers for response to weight loss, those for example might represent interesting candidates for further functional analyses. However, numerous further studies are needed to elucidate specific epigenetic fetal markers that are influenced by parental lifestyle (**Table 4**).

**Table 4:** *In-utero* methylation studies under lifestyle intervention.

Study	Participants	Methods	Results	References
antenatal diet/lifestyle intervention **Aim:** investigate the effect of an antenatal diet and maternal pre-pregnancy overweight or obesity, on infant cord blood DNA methylation	645 women with early pregnancy (BMI ≥25.0 kg/m^2^, mean age = 29.45)	Illumina HumanMethylation 450K Bead Chips Tissue: blood	No CpG sites were significantly differentially methylated in relation to either the diet and lifestyle intervention, or with maternal early pregnancy BMI	Louise et al. 2022 (92)
physical activity with or without dietary advice **Aim:** investigate whether a lifestyle intervention in pregnant women with obesity is associated with epigenetic variation in cord blood and body composition in the offspring	135 Obese Pregnant women (BMI = 34.10 kg/m^2^, Age = 30.90)	Illumina HumanMethylation 450K Bead Chips **Tissue:** blood	DNA methylation was altered at 379 sites, annotated to 370 genes following a lifestyle intervention versus control subjects; Methylation at 17 sites, *DISC1, GBX2, HERC2*, and *HUWE1*, partially mediates the effect of the lifestyle intervention on lean mass in the offspring	Jönsson et al. 2021 (89)
lifestyle intervention (low GI diet plus physical activity) **Aim:** investigate whether a dietary and physical activity intervention in pregnant women with obesity modified the methylation signatures associated with maternal dysglycaemia	557 pregnant women with obesity (BMI = 36.47±4.74 kg/m^2^, Age = 30.95 ± 5.42 years)	Illumina HumanMethylation 850K Bead Chips **Tissue:** blood	The most significantly GDM-associated CpG was cg03566881 located within *LGR6;* maternal dysglycaemia was associated with significant changes in the epigenome of the infants	Antoun et al. 2020 (90)
antenatal care plus a “Healthy Lifestyle Package” **Aim:** examine relationships between maternal glycaemia, insulinemic status, and dietary glycaemic indices during pregnancy	172 pregnant women who were overweight or obese (BMI = 29.78 ± 3.5 kg/m^2^, Age = 32.72 ± 4.5)	Illumina HumanMethylation 850K Bead Chips **Tissue:** blood	Insulin concentrations in late pregnancy were positively associated with DNAm changes at birth of the *RNF214* and *PCSK7* genes; Maternal indicators of insulin resistance and β-cell function in early pregnancy were associated with lower methylation near the *SYN3* and *JARID2* genes; maternal insulin sensitivity was associated with higher methylation on the *POLR2C* gene; no intervention effect on newborn DNAm,	Lecorguillé et al. 2022 (91)
Diet intervention **Aim:** investigated the impact of a low glycaemic index dietary intervention during pregnancy on offspring DNA methylation patterns	60 (30 Intervention group) neonates and womens (BMI = 27.72 kg/m^2^, Age = 32.78 years)	Illumina HumanMethylation 850K Bead Chips **Tissue:** blood	Widespread variation was identified in the newborns exposed to the dietary intervention; No association was found with maternal early-pregnancy body mass index (BMI), infant sex, or birth weight	Geraghty et al. 2018 (135)

This table summarizes our in-utero methylation studies under lifestyle intervention. BMI, body mass index; DISC1, Disrupted in schizophrenia 1 protein; GBX2, Gastrulation brain homeobox 2; HERC2, HECT and RLD domain containing E3 ubiquitin protein ligase 2; HUWE1, HECT, UBA and WWE domain containing E3 ubiquitin protein ligase 1; GI, glycaemic index; GDM, gestational diabetes mellitus; LGR6, Leucine rich repeat containing G protein-coupled receptor 6; DNAm, DNA-Methylation; RNF214, Ring finger protein 214; PCSK7, Proprotein convertase subtilisin/kexin Type 7; SYN3, Synapsin III; JARID2, Jumonji and AT-rich interaction domain containing 2; POLR2C, RNA Polymerase II subunit C.

### Future therapy options

Exploring causal epigenetic mechanisms in obesity could lead to novel treatments (138). There are already several pharmacological agents that affect DNA methylation and histone modifications, including DNMT inhibitors (e.g. Aza) and histone deacetylase inhibitors (HDACi, e.g. VPA and TSA). In addition, epigenetic drugs have already been tested for other diseases. This includes leukaemia, where Aza, BET and IDH1/2 inhibitors have been used in clinical trials (6, 113, 139, 140). Furthermore, some drugs such as metformin and statins, which are used to treat T2DM or lipid dysregulation in obesity, may affect epigenetic mechanisms (141–144). However, lifestyle interventions, including physical activity and a healthy diet, have been shown to be more effective than metformin in reducing the incidence of T2D in people at high risk (145), whereby lifestyle interventions continue to play an important role in elucidating epigenetic mechanisms and thus contributing to treatment options for obesity (**Figure 2**). On the other hand, DNA methylation marks could improve the success of weight loss therapies in the context of precision nutrition.

### Limitations

Due to the numerous elaborated limitations and factors influencing DNA methylation profiles, the results of the reviewed studies can only be assigned to biological mechanisms with caution. First, knowledge of large, sophisticated epigenome-based risk scores for nutritional interventions, as has already been done for genotype-based interventions (146–150), is essential. The lack of replication does not allow a definitive conclusion on genotype-diet interactions in weight loss, thus requiring further studies with larger sample sizes (151). Because research on longitudinal epigenetic changes is still a quite underrepresented area, most data come from observational studies only, and data on histone modifications and chromatin remodelling tend to be lacking (152). In addition, compared to a stable genotype which can alter individual's physiology and behaviour without active intervention (153), this review indicates that epigenetic modification are not only cause but mostly consequence of a lifestyle intervention, complicating the development of bioinformatics tools for personalized nutrition.

On the other hand, the gut microbiome has previously been reported to interact with the host gene expression and play causal roles in the development of several diseases, including obesity (154, 155) and diabetes (156). The composition of the microbiome changes rapidly up to the age of three, increases until around the age of 40 and then remains fairly stable (157–159). However, short-term modulations of the gut microbiome can disrupt normal metabolite production (84) and this can lead to changes in host gene expression, which in turn could trigger longer-lasting effects in the host. In addition, it has already been shown that the human microbiome can also influence epigenetic modifications such as DNA methylation and histone acetylation (85, 160). It is possible that the response to specific diet and exercise interventions is influenced by the microbiome, which could explain the high variability in individual response. However, increasing number of individual nutritional studies are now incorporating microbiome data obtained from stool samples by 16S RNA sequencing, and thus accelerating information about the interaction of the microbiome and host gene expression (152). As mentioned, another limitation is that the studies analyzed mostly blood samples and further studies examining lifestyle effects on epigenetic remodelling in metabolic target organs such as adipose tissue, liver and muscle are inevitable. In regard to both, the limitation of target tissue access and the ability of blood methylome mirroring target tissue changes, circulating cell-free DNA reveals a high potential to full this gap and serve as a direct proxy of e.g. AT specific methylation changes. Circulating cfDNAs are short double stranded DNA fragments with low concentrations in blood, urine and other fluids (161) which are able to maintain characteristics of the tissue of origin such as methylation, nucleosome confirmations and mutations (162, 163), making cfDNA to biomarker for disease detection (164, 165). However, although recent findings clearly indicate that cfDNA might also be involved in disease progression driven by obesity-related tissue degeneration (166, 167) (including insulin resistance (168)), it is not yet commonly used as a biomarker in metabolic diseases. Altogether, a subsequent meta-analysis based on identical bioinformatic data processing would be required to analyse and summarise the results of the included studies. However, meta-analyses are rarely represented in this research field, which might be driven through the high diversity between the different intervention studies and heterogeneity of the study cohorts. To overcome this problem, all studies would have to meet a certain standard in order to be comparable. These standards include high sample sizes with equal gender distribution, long-term study durations, and random assignment to intervention groups, i.e. randomised controlled trials.

## Conclusion

As demonstrated in the present review, the analysis of DNA methylation in a longitudinal design is very recent and the number of studies identified is excessively low to be able to draw relevant conclusions. Nevertheless, although the reviewed genome-wide studies are still lacking reproducibility, a number of candidate genes have been successfully validated. Recent studies on DNA methylation as weight loss predictor and mAge in relation to obesity state, further indicate its role and potential as an important biomarker for the improvement of obesity stratification and treatment.

## Author contributions

All authors listed have made a substantial, direct, and intellectual contribution to the work and approved it for publication.

## Glossary

**Table:**

A2MP1	Alpha-2-macroglobulin pseudogene 1
ACE2	Angiotensin converting enzyme 2
ADRB2	Adrenoceptor beta 2
AT	adipose tissue
AURKC	Aurora kinase C
BAG3	BAG cochaperone 3
BCAS4	breast carcinoma amplified sequence 4
BHMT2	betaine&ndash;homocysteine S-methyltransferase 2
BMI	body mass index
BWRP	body weight reduction program
C3orf38	chromosome 3 open reading frame 38
CAV1	Caveolin 1
CBFA2T3	CBFA2/RUNX1 partner transcriptional co-repressor 3
CLOCK	Circadian locomoter output cycles protein kaput
CRACR2A	Calcium release activated channel regulator 2A
CRISP2	Cysteine rich secretory protein 2
CRY2	Cryptochrome circadian regulator 2
DISC1	Disrupted in schizophrenia 1 protein
DMR	differentially methylated regions
DMP	differentially methylated position
EPDR1	ependymin related 1
EWAS	Epigenome-wide association studies
FARP1	FERM, ARH/RhoGEF and pleckstrin domain protein 1
FBXW5	F-Box and WD repeat domain containing 5
FDR	false discovery rate
FGFRL1	fibroblast growth factor receptor like 1
FKBP5	FKBP prolyl isomerase 5
FRMD4A	FERM domain containing 4A
FTO	Fat mass and obesity-related
GBP	gastric bypass
GBX2	Gastrulation brain homeobox 2
GDM	gestational diabetes mellitus
GDP	Guanosin diphosphate
GWAS	Genome-wide association studies
HERC2	HECT and RLD domain containing E3 ubiquitin protein ligase 2
HUWE1	HECT, UBA and WWE domain containing E3 ubiquitin protein ligase 1
IGHMBP2	immunoglobulin mu DNA binding protein 2
ITPR1	inositol 1, 4, 5-trisphosphate receptor type 1
JARID2	Jumonji and AT-rich interaction domain containing 2
JSRP1	junctional sarcoplasmic reticulum protein 1
KCNG2	potassium voltage-gated channel modifier subfamily G member 2
LEP	leptin
LGR6	Leucine rich repeat containing G protein-coupled receptor 6
LINC00539	Long intergenic non-protein coding RNA
LRFN4	leucine rich repeat and fibronectin type III domain containing 4
LRRC27	leucine rich repeat containing 27
mAge	methylation age
MAPK	mitogen-activated protein-kinase
MTSS1	Metastasis suppressor 1
MWAS	methylome-wide association study
MYH15	myosin heavy chain 15
NCOR2	nuclear receptor corepressor 2
NFATC2IP	Nuclear factor of activated T cells 2 interacting protein
NLRC3	NLR family CARD domain containing 3
NTSR1	Neurotensin receptor 1
NUDT3	nudix hydrolase 3
OSTM1	osteoclastogenesis associated transmembrane protein 1
PBMCs	Peripheral blood mononuclear cell
PCDHGA4	protocadherin gamma subfamily A, 4
PCR	polymerase chain reaction
PCSK7	Proprotein convertase subtilisin/kexin type 7
PER2	Period circadian regulator 2
POLR2C	RNA polymerase II subunit C
PON3	Paraoxonase 3
PTEN	Phosphatase and tensin homolog
Ras	Rat sarcoma
Rap1	Ras-related protein 1
RNF214	Ring finger protein 214
RNF39	Ring finger protein 39
SAT	subcutaneous adipose tissue
SH3PXD2A	SH3 and PX domains 2A
SLC6A12	Solute carrier family 6 member 12
SLFN12	Schlafen family member 12
SNP	Single nucleotide polymorphism
STK32C	Serine/threonine-protein kinase 32C
SULF2	Sulfatase 2
SYN3	Synapsin III
SYNJ2	synaptojanin 2
T2D	type 2 diabetes
TNMD	tenomodulin
TXNIP	Thioredoxin interacting protein
UCHL1	ubiquitin C-terminal hydrolase L1
VAT	visceral adipose tissue
VIPR2	vasoactive intestinal peptide receptor 2
VLCKD	very-low-calorie ketogenic diet
VWDE	von Willebrand factor D and EGF domains
WDPCP	WD repeat containing planar cell polarity effector
Wnt	wingless-type
ZNF331	zinc finger protein 331
